# Outcomes of thymoglobulin versus basiliximab induction therapies in living donor kidney transplant recipients with mild to moderate immunological risk – a retrospective analysis of UNOS database

**DOI:** 10.1080/07853890.2023.2215536

**Published:** 2023-05-26

**Authors:** Hatem Ali, Mahmoud Mohammed, Tibor Fülöp, Shafi Malik

**Affiliations:** a Renal Department, University Hospital Coventry & Warwickshire, Coventry, UK; b Medicine Department, North MS Medical Center, North Mississippi, MS, USA; c Department of Medicine - Division of Nephrology, Medical University Hospitals of SC, South Carolina, SC, USA; d Medicine Service, Ralph H. Johnson VA Medical Center, Charleston, SC, USA

**Keywords:** Induction therapy, renal transplant, kidney transplant, outcomes, high risk, HLA, PRA, living transplant

## Abstract

**Introduction:**

The aim of this study is to assess the outcomes of different induction therapies among mild to moderate immunological risk kidney transplants in the era tacrolimus and mycophenolate-derivate based maintenance.

**Methods:**

This was a retrospective cohort study using data from the United States Organ Procurement and Transplantation Network among mild to moderate immunological risk living-donor KTRs, defined as having first transplant and panel reactive antibodies less than 20% but with two HLA-DR mismatches. KTRs were divided into two groups based on induction therapy with either thymoglobulin or basiliximab. Instrumental variable regression models were used to assess the effect of induction therapy on acute rejection episodes, serum creatinine levels and graft survival.

**Results:**

Of the entire cohort, 788 patients received basiliximab while 1727 patients received thymoglobulin induction. There were no significant differences between basiliximab versus thymoglobulin induction in acute rejection episodes at one-year post-transplant (coefficient= −0.229, *p* value = .106), serum creatinine levels at one-year post-transplant (coefficient= −0.024, *p* value = .128) or death-censored graft survival (coefficient: − <0.001, *p* value = .201).

**Conclusion:**

This study showed no significant difference in acute rejection episodes or graft survival when using thymoglobulin or basiliximab in mild to moderate immunological risk living donor KTRs, maintained on tacrolimus and mycophenolate-based immunosuppressive regimen.

## Introduction

Human leukocyte antigen (HLA) matching and incorporating calculated panel reactive antibodies (C-PRA) have a substantial impact on the outcome of kidney transplantation [1]. While HLA mismatching represents an important prognostic factor, HLA-DR mismatching, in particular, has a greater impact on outcomes after kidney transplantation [2]. HLA-DR mismatching can increase the risk of developing donor-specific antibodies (DSA) and subsequently increase the risk of acute rejection episodes [1]. In a recent meta-analysis of 23 cohort studies, incremental HLA-DR mismatch was associated with worse transplant outcomes in terms of acute rejection as well as overall and death-censored graft survival [2]. C-PRA has been widely used a measure of sensitization among kidney transplant patients [3]. Since the mid-1960s, when it was discovered that catastrophic hyperacute rejection was linked to anti-donor HLA antibodies, PRA has been used to evaluate sensitization [4]. Using a panel of healthy blood donors as a representative sample of the possible local organ donor pool, Patel and Terasaki’s seminal study also presented a straightforward surrogate test that might identify sensitized patients and predict their likelihood of finding a crossmatch-compatible donor [4]. PRA was only the portion of this pool of donors that a patient’s reactive antibodies were present against. Crossmatch incompatibility for a patient with an 80% PRA would be with 80% of donors. A calculated PRA of 20% has been used as a cut-off point for mild immunological risk by many transplant centres [5,6]. A combination of HLA mismatch and C-PRA can be used to assess the immunological risk in transplant.

The recommendations for using thymoglobulin ([rabbit-derived] polyclonal anti-thymocyte globulin) induction therapy are based on the results of a previous meta-analysis that compared basiliximab and thymoglobulin induction therapies in kidney transplant patients [7]. However, the maintenance immunosuppression in most of the reviewed studies was in the era of cyclosporine-based immunomodulating therapy. Currently, most transplant centres depend on tacrolimus as an efficacious cornerstone immunosuppressant in kidney transplantation [8]. Many randomized, multicentre studies conducted in Europe and the US with long follow-up periods showed a significantly lower incidence of acute rejection and improved survival in renal transplant recipients receiving tacrolimus-based immunosuppression compared to those receiving cyclosporine [9,10]. This raises the question of whether basiliximab can be an effective induction therapy in mild to moderate immunological risk kidney transplant patients maintained on tacrolimus or not. Living-related transplant recipient have minimized cold ischaemia times, affording elimination one of the largest variables impacting allograft survival and affording a much cleaner clinical model to assess impact of immunologic incompatibilities and applied therapies. Thus, we aimed to examine the outcomes of basiliximab induction therapy in comparison to thymoglobulin induction therapy in mild to moderate immunological risk living donor kidney transplant recipients (KTRs) maintained on tacrolimus and mycophenolate-based immunomodulating therapy.

## Methodology

### Design and study cohort

Because the initiative used publicly available, de-identified data, it was exempt from institutional approval. There was no financial assistance received for this study. Terminology and nomenclature were expressed in keeping with most recent KDIGO consensus guidelines [11]. Data for this study is publicly available in a de-identified fashion and can be accessed through: https://optn.transplant.hrsa.gov. All renal transplant patients who were registered in the United States Organ Procurement and Transplantation Network (OPTN) database between the first of September 2017 and the first of September 2019 were retrospectively reviewed. 2017 was chosen to open the review window due to being the year the U.S. Food and Drug Administration (FDA) statement confirmed the use of thymoglobulin as induction therapy for renal transplantation with specific doses recommended [12].

The patients included were all living donor KTRs with mild to moderate immunological risk who received thymoglobulin or basiliximab induction therapy and were discharged on tacrolimus and mycophenolate mofetil as a maintenance immunosuppressive therapy. Mild to moderate immunological risk kidney transplant was defined as living donors who received their first transplant with PRA less than 20% and had two HLA-DR mismatch. Exclusion criteria applied to patients with previous kidney transplants, those under 18 years of age, deceased donors organ recipients, patients whose DR mismatch was less than two, patients who received an induction therapy other than thymoglobulin or basiliximab, patients who received maintenance immunosuppressive medications other than tacrolimus and mycophenolate mofetil, patients who received both thymoglobulin and basiliximab at the same time and those who had missing data regarding their induction therapy. Patients were followed up until December 2020. Data were collected about recipient factors (recipient age, gender, ethnicity, body mass index), transplant factors (cold ischaemia time, number of previous transplants, calculated panel reactive antibodies, HLA mismatches, type of induction therapies, maintenance immunosuppressive medications) and donor factors (donor type, donor age). Based on the induction therapies administered, kidney transplant recipients were divided into two groups: thymoglobulin or basiliximab therapy recipients.

### Main outcomes

The primary outcomes measured were the occurrence of acute rejection episodes at the early post-operative period as well as one-year post-transplant and serum creatinine levels at one -year post-transplant. Acute rejection was defined as biopsy-proven or clinically suspected rejection episodes. Secondary outcomes were the occurrence of delayed graft functions (defined as the need for regular dialysis during the first week following transplantation), overall and death-censored graft survival.

### Statistical analysis

The study groups were compared based on baseline characteristics. Continuous variables were compared using the two-Independent T-test and categorical variables were compared using Pearson’s chi-squared test. Ten events per variable was the cut-off point to proceed with the regression analysis. Instrumental variable-ordered ‘probit’ regression analysis was used to assess the relationship between the type of induction therapy and the occurrence of acute rejection episodes at one-year post-transplant. The model was adjusted for recipient, transplant and donor factors collected. The type of induction therapy was instrumented for the transplant centre to reduce the centre effect on the choice of induction therapy. The choice of instrumenting the type of induction therapy to the transplant centre was based on the hypothesis that in the current era, immunosuppressive regimens are protocol-driven and differ from one centre to another [13]. We used the Wald test to assess for exogeneity. The Wald test measures the correlation between the error terms in the probit regression and the instrumented regression. A *p* value of ≤.05 was the cut-off point to reject the null hypothesis for no endogeneity.

To assess the relationship between induction therapy and serum creatinine at one-year post-transplant, we performed the instrumental variable linear regression model. The estimator is used as a generalized method of moments. The type of induction therapy was instrumented for the transplant centre to reduce the centre effect on the choice of induction therapy. We used the GMM C-Statistics to assess for exogeneity. A *p* value of ≤.05 was the cut-off point to reject the null hypothesis of no endogeneity.

To perform the analysis for overall and death-censored graft survival, considering the centre effect on the choice of induction therapy, we generated pseudo-observations for the survival function. These pseudo-observations were used in a generalized linear model. In these models, the type of induction therapy was instrumented for the transplant centre to reduce the centre effect on the choice of induction therapy. We generated pseudo-observations for the survival function using the ‘STPSURV’ command [14]. The generation of pseudo-observations is a method that has been developed to use survival function in direct regression and generalized linear modelling [9, 10]. The pseudo-observations are calculated based on the difference between the complete sample and leave-one-out estimators for the pertinent survival quantity [14, 15]. The pseudo-observations have been proven to give tight approximation to the Cox regression models.

To assess the relationship between the occurrence of delayed graft function and the type of induction therapy, we applied the approach used for the assessment of acute rejection episodes.

### Sensitivity analysis

We performed a sensitivity analysis for the relationship between induction therapies and estimated the doses of thymoglobulin and basiliximab for the available data using the number of days each induction therapy was administered. In April 2017, the FDA statement confirmed the use of Thyroglobulin as induction therapy for renal transplantation. It set the dose at 1.5 mg/kg dose with an administration period of up to seven days post-transplant [12]. It also determined the dose of basiliximab to be 20 mg/kg for two doses to be administered at day 0 and day 4 post-transplant. Based on this, we estimated the overall dose of the induction therapy given by multiplying the number of days it was administered by the approved dose stated by the FDA. We performed an instrumental variable-ordered probit regression analysis, to compare different doses of thymoglobulin and basiliximab. We also performed a multivariable logistic regression analysis to assess the relationship between acute rejection and induction therapies without considering the centre effect. Details of the logistic regression analysis are discussed in the Supplementary data section. Furthermore, we repeated the logistic regression model among several subgroups (black population, non-black population, patients discharged on glucocorticoid withdrawal regimen, male donor to female recipient and female donor to male recipient).

## Results

**Figure 1.**
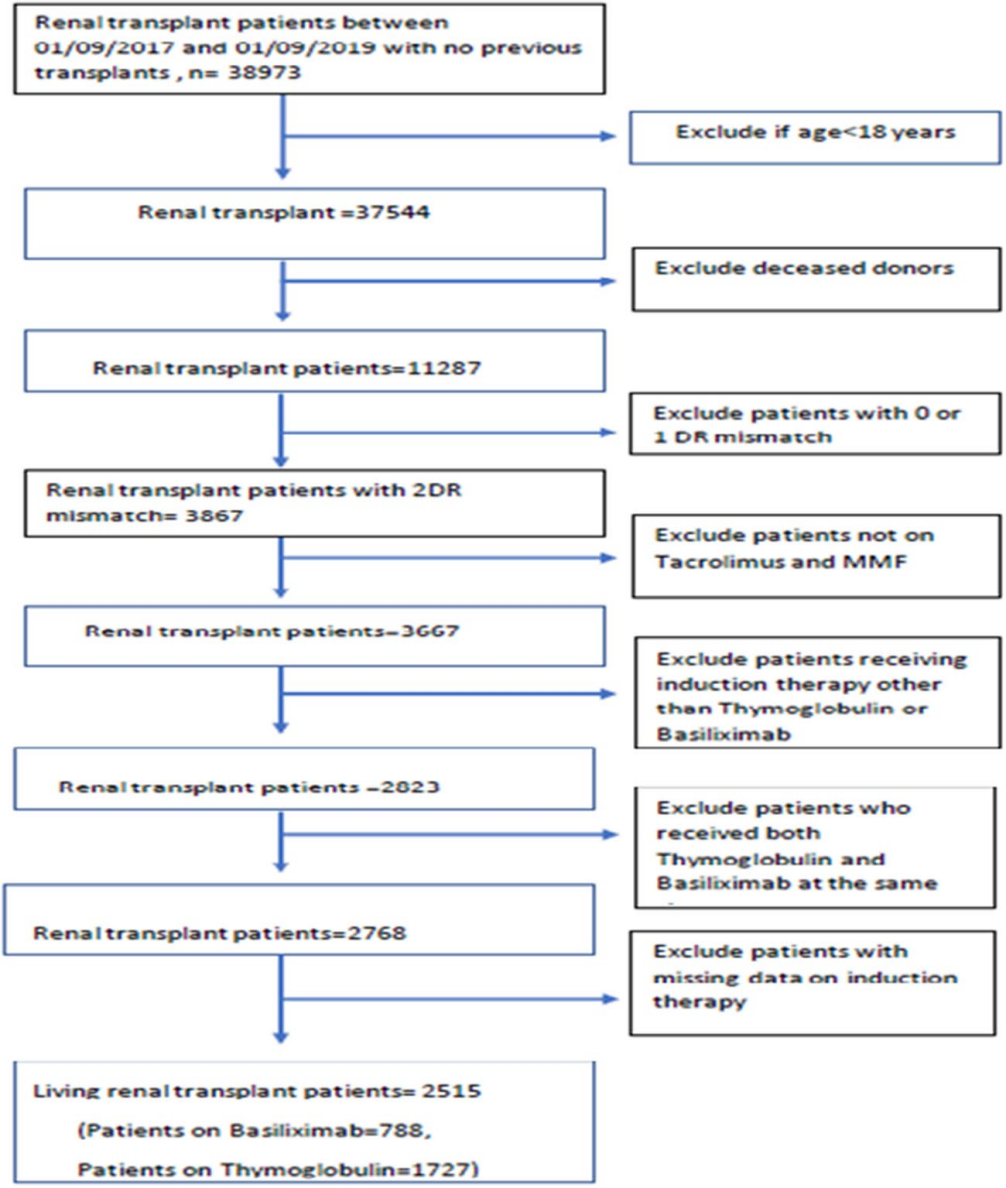
Details of patient selection from the OPTN database.

A total of 2515 patients were included in our study (patients on basiliximab = 788, patients on thymoglobulin = 1727). The details of patient selection from the OPTN database are shown in Figure 1. The baseline characteristics for the patients included in our study are shown in Table 1. Data concerning the type of induction therapy given were missing in the case of 246 patients. The comparison between the baseline characteristics of those with missing data versus those with no missing data is shown in Table S1 of the Supplementary data section. There were no significant differences between the two groups, except in HLA-A mismatch (*p* = .04) and frequency of glucocorticoid maintenance therapy (*p* value <.01). None of the patients included had positive crossmatch.

**Table 1.** Baseline characteristics of study groups.

	Thymoglobulin group (1727)	Basiliximab group (788)	*p* Value
Recipient age in years: mean (standard deviation)	49.51 (13.36)	55.14 (14.21)	<.01
Recipient body mass index (BMI): mean (standard deviation)	28.52 (5.48)	28.43 (5.32)	.72
Recipient sex: male (n,%)	1087 (62.94%)	549 (69.67%)	<.01
Recipient diabetes:	467 (27.04%)	260 (32.99%)	<.01
Yes (n, %)			
Ethnicity (n,%):			<.01
White	1107 (64.09%)	535 (67.89%)	
Black	273 (15.80%)	76 (9.64%)	
Hispanic	203 (11.75%)	101 (12.81%)	
Asian	112 (6.48%)	61 (7.74%)	
American Indian	9 (0.52%)	5 (0.63%)	
Pacific Islander	2 (0.11%)	3 (0.38%)	
Multiracial	21 (1.21%)	7 (0.88%)	
PRA, % : mean (standard deviation)	10.22 (21.67)	5.09 (14.97)	<.01
Cold ischaemia time in hours: mean (standard deviation)	2.61 (4.17)	2.81 (4.76)	.29
HLA-A mismatch (N,%):			.60
0	135 (7.81%)	54 (6.85%)	
1	752 (43.54%)	338 (42.89%)	
2	840 (48.63%)	396 (50.25%)	
HLA-B mismatch : (N,%)			.22
0	29 (1.67%)	12 (1.52%)	
1	401 (23.21%)	208 (26.39%)	
2	1297 (75.10%)	568 (72.08%)	
Glucocorticoid maintenance regimen (n,%):	1127 (65.33%)	671 (85.15%)	<.01
Donor age: mean (standard deviation)	44.73 (12.15)	47.82 (12.83)	<.01
Donor type:			
Unrelated (n,%)	1510 (87.43%)	685 (86.92%)	.72
Donor sex: male (n,%)	590 (34.16%)	259 (32.86%)	.52
Days of induction therapies	1 day:36 2 days: 155 3 days: 918 4 days 484 5 days 98 6 days: 3 7 days 2 8 days : 3	2 days: 788	

### Primary outcomes

#### Acute rejection rates at one-year post-transplant

Among the 2515 patients included in our study, 2058 patients had available data about acute rejection rates at one-year post-transplant, while 457 (18.17%) patients had missing data about acute-rejection episodes. Comparison between patients with missing data versus those with no-missing data are shown in Table S2 of Supplementary data section.

On performing an instrumental variable - ordered probit regression analysis, there was no statistical difference between thymoglobulin induction therapy and basiliximab induction therapy (coefficient= −0.229, *p* value = .106, 95% Confidence interval [95% CI]: −0.508 to 0.049), as shown in Table 2. The Wald test for exogeneity showed a *p* value of .044, rejecting the null hypothesis for no endogeneity. The number of events in the basiliximab and thymoglobulin groups was 44 and 116, respectively.

**Table 2.** Instrumental variable-ordered probit regression analysis assessing the relationship between induction therapy and acute rejection episodes at 12 months post-transplant.

	Coefficient	Standard error	*p* Value	95% Confidence interval
Induction therapy (basiliximab versus thymoglobulin)	−0.229	0.142	.106	−0.508 to −0.049
Sex				
Male	0.019	0.093	.983	−0.181 to 0.185
Body mass index	0.006	0.007	.375	−0.008 to 0.022
Cold ischaemia time	0.002	0.010	.777	−0.016 to 0.022
Serum creatinine at discharge	0.070	0.029	.016	0.013 to 0.127
Calculated PRA	− <0.001	0.002	.866	−0.004 to 0.003
Ethnicity				
Black	0.017	0.120	.887	−0.219 to 0.253
Hispanic	−0.166	0.138	.229	−0.438 to 0.104
Asian	−0.554	0.230	.016	−1.006 to −0.102
American Indian	0.053	0.536	.920	−0.998 to 1.105
Pacific Islander	−4.814	3005.509	.999	−5895.504 to 5885.875
Multiracial	−4.556	1293.668	.997	−2540.099 to 2530.987
Recipient age	−0.014	0.003	<.001	−0.021 to −0.006
HLA-A mismatch	0.061	0.710	.390	−0.078 to 0.200
HLA-B mismatch	−0.092	0.086	.285	−0.549 to −0.187
Glucocorticoid maintenance	−0.368	0.0924	<.001	−0.549 to −0.187
Donor age	0.014	0.004	.001	−0.005 to 0.224

#### Serum creatinine at one-year post-transplant

On performing an instrumental variable-linear regression analysis, there was no statistical difference between thymoglobulin induction therapy and basiliximab induction therapy (coefficient= −0.024, *p* value = .128, 95% CI: −0.055 to 0.006), as shown in Table 3. GMM C-statistics showed a *p* value of .049, rejecting the null hypothesis for no endogeneity. F-Statistics showed a *p* value of <.01, rejecting the null hypothesis for weak instruments. Mean serum creatinine in the basiliximab group was 1.35 mg/dl (Standard deviation = 0.41) and 1.37 mg/dl (Standard deviation = 0.47) in the thymoglobulin group.

**Table 3.** Instrumental variables linear regression model assessing the relationship between serum creatinine at one-year post-transplant and induction therapy.

	Coefficient	Robust standard error	*p* Value	95% Confidence interval
Induction therapy (basiliximab versus thymoglobulin)	−0.024	0.015	.126	−0.054 to 0.006
Sex				
Male	0.238	0.013	<.001	0.211 to 0.265
Body mass index	0.002	0.001	.019	<0.001 to 0.005
Cold ischaemia time	−0.004	0.001	<.001	−0.006 to −0.001
Serum creatinine at discharge	0.089	0.010	<.001	0.068 to 0.109
Calculated PRA %	<0.001	<0.001	.220	− <0.001 to 0.001
Ethnicity				
Black	0.111	0.021	<.001	0.070 to 0.153
Hispanic	−0.049	0.018	.009	−0.086 to −0.012
Asian	−0.136	0.023	<.001	−0.182 to −0.089
American Indian	−0.017	0.061	.783	−0.138 to 0.104
Pacific Islander	0.008	0.082	.917	−0.153 to 0.170
Multiracial	0.008	0.047	.861	−0.084 to 0.101
Recipient age	−0.004	<0.001	<.001	−0.005 to −0.003
HLA-A mismatch	0.008	0.010	.410	−0.011 to 0.028
HLA-B mismatch	−0.004	0.012	.741	−0.028 to 0.020
Glucocorticoid maintenance	−0.041	0.014	.005	−0.070 to −0.012
Donor age	0.007	<0.001	<.001	.006 to 0.008

#### Secondary outcomes

There was no statistical difference between the two groups in terms of overall graft survival (coefficient = 0.008, *p* value = .801, 95% CI: −0.001 − 0.001), as shown in Figure 1 in the Supplementary data section. There was no statistical difference between the two groups in terms of death-censored graft survival (coefficient: − <0.001, 95% CI: −0.001 − 0.001, *p* value = .201), as shown in Figure S2 in the Supplementary data section. The median follow-up was one-year post-transplant.

#### Sensitivity analysis

We performed an instrumental variable-ordered probit regression analysis, to compare the different doses of thymoglobulin and basiliximab and its relation to acute rejection episodes at one year post-transplant. There was no statistical difference between basiliximab and two days of thymoglobulin (estimated dose = 3 mg/kg, coefficient = 0.528, *p* value = .763, 95% CI: −2.910 to 3.967), three days of thymoglobulin (estimated dose = 4.5 mg/kg, coefficient= −0.427, *p* value = .507, 95% CI: −1.690 to 0.835), four days of thymoglobulin (estimated dose = 6 mg/kg, coefficient= −0.226, *p* value = 1) or five days of thymoglobulin (estimated dose = 7.5 mg/kg, coefficient = 0.009, *p* value = .997, 95% CI: −5.111 to 5.129). Additionally, we also performed a multivariable logistic regression analysis to assess the relationship between acute rejection and induction therapies without taking into account the centre effect. This showed no statistical difference between basiliximab induction therapy and thymoglobulin induction therapy (OR = 0.934, *p* value = .737, 95%CI: 0.628–1.388), as shown in Table S3 in the Supplementary data section. Moreover, there was no statistical difference basiliximab induction therapy and thymoglobulin induction therapy among black population subgroup, non-black population subgroup, or steroids withdrawal subgroup, male donor to female recipient or female donor to male recipient as further shown in Table S4 of Supplementary data.

## Discussion

In tacrolimus-maintained living donor kidney transplant recipients with mild to moderate immunological risk, we found no significant differences between basiliximab and thymoglobulin induction therapies in terms of acute rejection episodes, serum creatinine or graft survival at one-year post-transplant. Our study can aid to accurately interpret prior experiences and to specify the application of various induction agents by including them into contemporary pretransplant immunological risk assessment methodologies.

The patient’s immunological risk status should be taken into consideration when choosing a regimen, and immunosuppression should be tailored to the risk for graft rejection unless there are obvious risk factors for drug-specific adverse effects. However, even though a patient’s risk status may be influenced by a variety of variables, only the number of HLA mismatches has been consistently associated with an increase in risk, and the relative significance of other variables frequently remains ambiguous. A recent study by Sureshkumar et al. compared the effect of induction therapy using depleting agents versus IL-2RA in more than 63,000 patients in the USA between 2001 until and 2015 [16]. They stratified the patients according to HLA-DR mismatches into three groups (zero, one and two mismatches) with a median follow-up period of 49 ± 62 months. They found that depleting antibodies are associated with better patient or graft survival in comparison to non-depleting antibodies. However, the calcineurin inhibitor agent used as maintenance therapy in this study was not specified. In the previous decade (2000–2010), cyclosporine use was still prevalent as a maintenance therapy, which is not the case in the current era, when most centres use tacrolimus. Moreover, the depleting antibody group could have received either alemtuzumab or thymoglobulin, further limiting the study’s current relevance.

HLA matching has a vital effect on the outcome of kidney transplantation [17]. While total HLA mismatching represents an important prognostic factor [16,17], HLA class II, especially HLA-DR mismatching has a greater impact on outcomes after kidney transplantation [2]. This is due to the high polymorphism in HLA Class II antigens. HLA class II antigens are present both in B-cells and antigen-presenting cells (APCs), which play a crucial role in the development of acute cell-mediated and antibody-mediated rejection. These antigen-presenting cells engulf process antigens and stimulate CD4 T-cells. The high polymorphism characteristic of Class II HLA antigens present on the APCs plays a pivotal role in identifying a large repertoire of foreign antigens. On the other hand, this high polymorphism acts as a barrier against successful transplantation, identifying allograft antigens as foreign antigens and stimulating rejection. Data from large registry studies have shown an approximately 7–13% higher risk of graft failure associated with one HLA mismatch and about 64–74% higher risk associated with six HLA mismatches [2, 18] with HLA-DR matching having a much greater effect on the number of rejection episodes and poor long-term survival [19, 20].

The initial Collaborative Transplant Study (CTS) analysis revealed that the major effect on transplant outcomes arose from mismatches in the HLA-DR [21, 22]. This was also noted in registry data studies performed using The United Kingdom Transplant Service and Eurotransplant registry data [23, 24]. Another study by Coupel et al. found that HLA-DR mismatches (and the number of rejection episodes) correlated with poor long-term survival [21]. Several immunosuppressive protocols have been implemented to overcome the detrimental effects of HLA mismatches. In the current era, tacrolimus and mycophenolate derivates (mycophenolate mofetil or enteric-coated mycophenolate sodium) are the core maintenance immunosuppressive therapies used worldwide. Thymoglobulin and basiliximab are the most widely used induction therapies [7, 8]. Thymoglobulin is usually reserved for high-risk transplants; however, it is not free of risk. It carries a higher risk of malignancy and serious opportunistic infections in comparison to basiliximab induction therapy [20].

Studies that were performed in cyclosporine era showed significant effect of HLA mismatching, particularly HLA-DR mismatch on graft outcomes [2]. In our study, the effect of HLA-DR mismatching is minimized, reflecting the effect of more potent immunosuppressive therapy to improve graft tolerance. Our study adds robustness and reflects the potency of tacrolimus-based immunomodulating therapy over cyclosporine-based regimens.

We used the available data from the UNOS database to conduct our study, which data has implicit internal limitations. The UNOS database record data about only low-resolution HLA-mismatch and therefore, mismatching at epitope or eplet levels cannot be excluded. With the most current technology, it has been shown that mismatching at the epitope or eplet level can confer a significant risk on transplant outcomes [25, 26]. Low-resolution data on donor and recipient HLA types is insufficient to fully realize the promise of better matching, although it does have some prognostic utility. In addition, the UNOS database does not reveal mismatches at other types of Class II HLA antigens, especially the HLA DQ antigen. Several studies have shown that an HLA DQ mismatch can lead to worse outcomes in comparison to matched HLA DQ transplants, irrespective of the HLA DR mismatch [27, 28]. Moreover, there was significant difference in PRA levels between basiliximab and thymoglobulin groups. However, the PRA levels in both groups were less than 10%, which is considered as the cut-off for standard immunological risk transplants in most of the literature [5, 9]. In addition, we included patients with no previous transplants in our study. Therefore, the risk of sensitization due to previous transplant is effectively eliminated.

Furthermore, there was a statistical difference between both induction groups in terms of donor age. However, from the clinical point of view, both ages (44.73 in the thymoglobulin group and 47.82 in the basiliximab group) are very similar and do not meet the criteria for expanded criteria donor [5, 13]. Our results reflect that selection of living donors with age less than 50 leads to acceptable transplant outcomes irrespective of HLA mismatching.

Notwithstanding the disparities in glucocorticoid withdrawal, PRA level and percentage of African American ethnicity between the thymoglobulin group and the Basiliximab group, the findings of our study can help clinicians decide which induction therapy is best for a transplant patient. In the event that a person is of African ancestry and has a greater PRA (even more than 0%), most clinicians advise employing thymoglobulin induction. Glucocorticoid withdrawal is not advised if basiliximab induction therapy is being used.

Another limitation of our study was the missing data about tacrolimus trough levels. Therefore, we cannot assess the difference in tacrolimus trough levels between both groups. In addition, calculation of thymoglobulin dose may not be accurate since some patients may have received less than 1.5 mg/d daily dose due to low white blood cells count and thrombocytopenia. Taking into account the limitations of our study and the retrospective nature of the UNOS database, we recommend that a randomized case-control study should be performed to confirm our results.

Our data analysis is also limited by the ongoing developments in transplant immunology. First of all, there is growing interest in using DSA for risk assessment [13]. Unfortunately, DSA results are unmeasured confounders in the registry database. DSA tests are one of the new technologies that are used to determine immunological risk but are not used or available worldwide, e.g. many countries in Africa and Asia are not using molecular methods such as Luminex. More studies are needed to identify immunological risk based on DSA.

Second, the development of solid-phase single-bead antigen testing of solubilized human leukocyte antigens (HLA) to detect donor-specific antibodies (DSA) has made it possible to stratify immunological risk status in a much more nuanced manner, taking into account the various classes and intensities of HLA antibodies Class I and/or II, including HLA-DSA [13]. Combinations of these tests are now frequently used to evaluate immunologic risk, with further technological developments emerging, such as the detection of non-HLA antibodies against angiotensin type 1 (AT1) receptors or the T-cell ELISPOT assay of alloantigen-specific donors [29]. Retrospective data analysis of UNOS (or any other administrative or clinical database) is typically intended to help understanding of past clinical practice and provide support for future improvement of results. However, even with regard to potential future developments in data recording and registry content, properly analysing and interpreting existing data can undoubtedly advance research. Finally, the black population is under-represented in our study. Our subgroup analysis showed no significant differences between both types of induction therapy among black population.

In conclusion, within the limitations of UNOS database, our study showed no significant difference in acute rejection episodes or graft survival when using thymoglobulin or basiliximab in mild to moderate immunological risk living donor KTRs. Therefore, in the current era of tacrolimus and mycophenolate agent-based maintenance immunosuppression, basiliximab may be a safe induction therapy for this class of recipients. Tailoring the induction therapy in kidney transplant patients should be adjusted based on the patients’ needs and reflected in institutional protocols.

## Supplementary Material

Supplemental Material

Supplemental Material

Supplemental Material

## Data Availability

Data is available in the Organ Procurement and Transplantation Network (OPTN) database. Data can be accessed through: https://optn.transplant.hrsa.gov. Code availability: STATA Package 17 was used for statistical analysis.
