# Supplementary figures and images for: Outcomes of thymoglobulin versus basiliximab induction therapies in living donor kidney transplant recipients with mild to moderate immunological risk – a retrospective analysis of UNOS database
Source: Ann Med. 2023 May 26;55(1):2215536. doi: 10.1080/07853890.2023.2215536 (PMC10228322; doi:10.1080/07853890.2023.2215536)

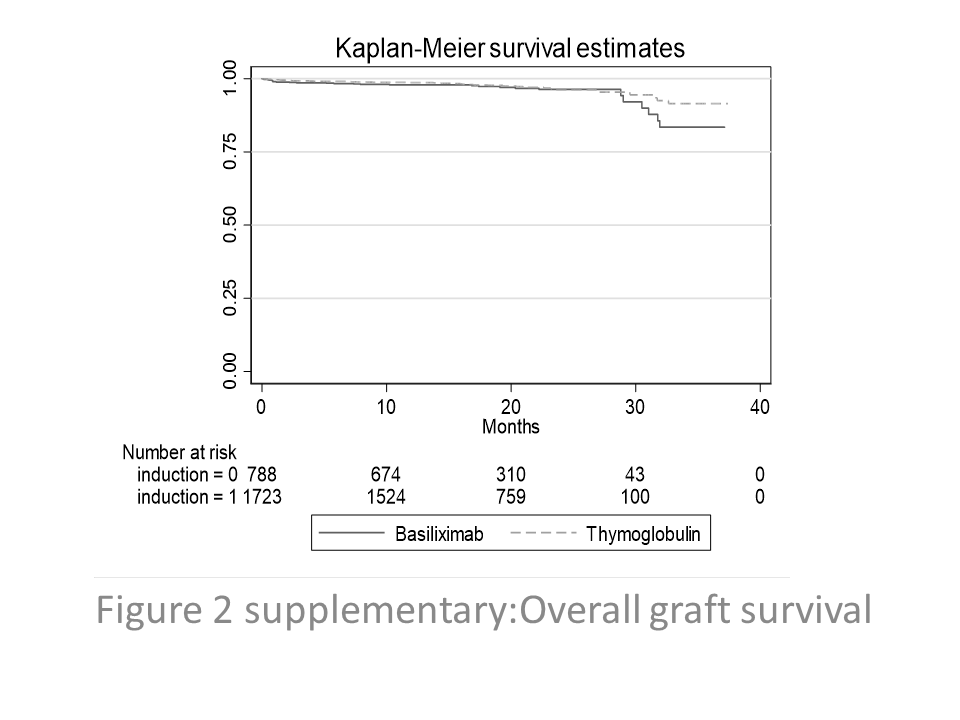

Supplement: Supplemental Material [file IANN_A_2215536_SM0049.tif]

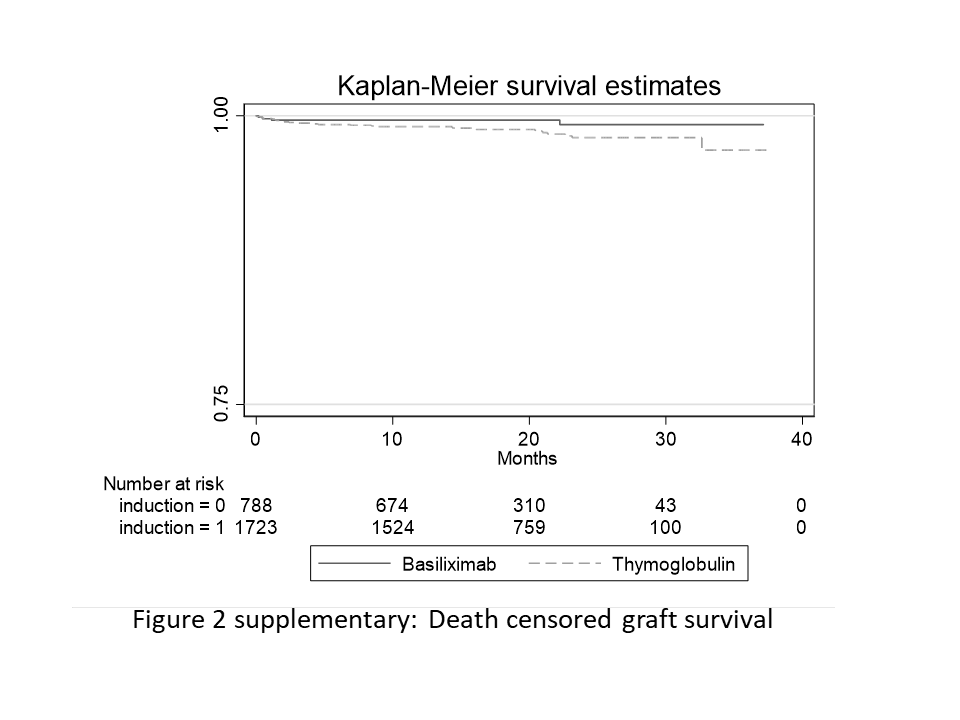

Supplement: Supplemental Material [file IANN_A_2215536_SM0030.tif]
