# Supplementary material for: Outcomes of thymoglobulin versus basiliximab induction therapies in living donor kidney transplant recipients with mild to moderate immunological risk – a retrospective analysis of UNOS database
Source: Ann Med. 2023 May 26;55(1):2215536. doi: 10.1080/07853890.2023.2215536 (PMC10228322; doi:10.1080/07853890.2023.2215536)
Supplement: Supplemental Material [file IANN_A_2215536_SM0020.docx]

|  | Non-missing data (2515) | Missing data (246) | P-value |
| --- | --- | --- | --- |
| Recipient age in years: mean (standard deviation) | 51.27 (13.87) | 50.36 (13.43) | 0.32 |
| Sex: male (n,%) | 1636 (65.04%) | 158 (64.22%) | 0.79 |
| Ethnicity (n,%):  White  Black  Hispanic  Asian  American Indian  Pacific Islander  Multiracial | 1642 (65.28%)  349 (13.87%)  304 (12.08%)  173 (6.87%)  14 (0.55%)  5 (0.19%)  28 (1.11%) | 172 (69.91%)  23 (9.34%)  32 (13.00%)  15 (6.09%)  1 (0.40%)  1 (0.40%)  2 (0.81%) | 0.51 |
| PRA : mean (standard deviation) | 8.61 (19.96) | 8.45 (19.31) | 0.90 |
| Cold ischemia time in hours: mean (standard deviation) | 2.67 (4.37) | 2.68 (4.10) | 0.94 |
| HLA-A mismatch : mean (standard deviation) | 1.41 (0.62) | 1.50 (0.59) | 0.04 |
| HLA-B mismatch : mean (standard deviation) | 1.72 (0.48) | 1.71 (0.46) | 0.85 |
| Glucocorticoid maintenance regimen (n, %): | 1798 (71.49%) | 195 (79.26%) | <0.01 |
| Donor age: mean (standard deviation) | 45.69 (12.44) | 45.04 (12.16) | 0.49 |

Table 1 Supplementary data: Comparison between patients with missing data about induction therapy versus patients included in the study.

|  | Non-missing data about acute rejection at one-year post-transplant (2058) | Missing data about acute rejection at one-year post-transplant (457) | P-value |
| --- | --- | --- | --- |
| Recipient age in years: mean (standard deviation) | 51.20 (13.57) | 51.62 (15.19) | 0.55 |
| Sex: male (n,%) | 1334 (64.82%) | 302(66.08%) | 0.60 |
| Ethnicity (n,%):  White  Black  Hispanic  Asian  American Indian  Pacific Islander  Multiracial | 1333 (64.77%)  290 (14.09%)  250 (12.14%)  149 (7.24%)  13 (0.63%)  4 (0.19%)  19 (0.92%) | 309 (67.61%)  59 (12.91%)  54 (11.81%)  24 (5.25%)  1 (0.21%)  1 (0.21%)  9 (1.96%) | 0.42 |
| PRA : mean (standard deviation) | 8.88 (20.05) | 7.41 (19.49) | 0.15 |
| Cold ischemia time in hours: mean (standard deviation) | 2.64 (4.38) | 2.81 (4.32) | 0.44 |
| HLA-A mismatch : (n,%)  0  1  2 | 147 (7.14%)  890 (43.24%)  1021 (49.61%) | 42 (9.19%)  200 (43.76%)  215 (47.04%) | 0.27 |
| HLA-B mismatch : (n,%)  0  1  2 | 31 (1.50%)  496 (24.10%)  1531 (74.39%) | 10 (2.18%)  113 (24.72%)  334 (73.08%) | 0.54 |
| Glucocorticoid maintenance regimen (n, %): | 1441 (70.01%) | 357 (78.11%) | <0.01 |
| Donor age: mean (standard deviation) | 45.39 (12.30) | 47.06 (13.01) | <0.01 |

Table 2 Supplementary data: Comparison between patients with missing data about acute rejection versus patients with no missing data.

|  | Odds Ratio | Standard error | P-value | [95% Conf.  Interval] |
| --- | --- | --- | --- | --- |
| Basiliximab versus Thymoglobulin | 0.934 | 0.188 | 0.737 | 0.628 to 1.388 |
| Donor age (years) | 1.028 | 0.008 | 0.001 | 1.011 to 1.046 |
| Glucocorticoid maintenance (yes) | 0.510 | 0.091 | <0.001 | 0.358 to 0.726 |
| Recipient age (years) | 0.972 | 0.007 | <0.001 | 0.958 to 0.987 |
| Ethnicity  White  Black  Hispanic  Asian  Pacific Islander  Multiracial | 1.126  0.722  0.318  1.182  1  1 | 0.262  0.206  0.165  1.247 | 0.609  0.255  0.028  0.874 | 0.713 to 1.779  0.412 to 1.264  0.114 to 0.884  0.149 to 9.353 |
| Serum creatinine at discharge (mg/dL) | 1.146 | 0.056 | 0.006 | 1.040 to 1.263 |
| HLA-A mismatch | 1.148 | 0.163 | 0.331 | 0.868 to 1.519 |
| HLA-B mismatch | 0.853 | 0.147 | 0.360 | 0.608 to 1.197 |
| Calculated PRA % | 1.0003 | 0.004 | 0.931 | 0.991 to 1.009 |
| Cold ischemia time (hours) | 1.007 | 0.019 | 0.712 | 0.970 to 1.045 |

Table3 Supplementary data:

Multivariable logistic regression analysis to assess the relationship between induction therapies and acute rejection rates at one-year post-transplant. We performed a univariable logistic regression analysis and assessed the relationship between donor, recipient, transplant factors and the risk of acute rejection. Variables with a p-value lower than 0.25 were included in the multivariable analysis. We used a backward stepwise approach to perform the logistic regression analysis. We used the Scaled Schoenfeld residuals to assess for proportionality assumption for each variable and for the whole model. The following variables were included as a PRIORI in the multivariable analysis due to their direct and well-known relationship with the occurrence of rejection: HLA-A mismatch, HLA-B mismatch and cold ischemia. Other variables that had a p-value higher than 0.25 were excluded from the model in a backward stepwise approach in order to achieve the best fit for the model. When comparing the basiliximab induction therapy to different doses of Thymoglobulin, there was no significant difference between the two groups (coefficient=1).

|  | Odds ratio | P value | 95%-confidence interval |
| --- | --- | --- | --- |
| ^a-^Black population | 0.47 | 0.12 | 0.18 to 1.23 |
| ^b-^Non-Black population | 1.21 | 0.37 | 0.78 to 1.86 |
| ^c-^Glucocorticoid withdrawal | 1.19 | 0.64 | 0.55 to 2.58 |
| ^d-^Male donor to female recipient | 1.05 | 0.93 | 0.30 to 3.64 |
| ^e-^Female donor to male recipient | 1.17 | 0.57 | 0.66 to 2.05 |

Table 4 Supplementary: Relationship between induction therapy and acute rejection episodes at one-year post-transplant among:

a: Black population

b: Non-black population

c: Glucocorticoid withdrawal

d: Male donor to female recipient

e: Female donor to male recipient
